# Fidelity and Clinical Competence in Providing Illness Management and Recovery: An Explorative Study

**DOI:** 10.1007/s10597-023-01137-7

**Published:** 2023-05-30

**Authors:** Bert-Jan Roosenschoon, Jaap van Weeghel, Mathijs L. Deen, Emmie W. van Esveld, Astrid M. Kamperman, Cornelis L. Mulder

**Affiliations:** 1 Department of Psychiatry, Epidemiological and Social Psychiatric Research Institute, Erasmus MC University Medical Centre, Doctor Molewaterplein 40, Rotterdam, 3015 GD The Netherlands; 2 Parnassia Academy, Parnassia Psychiatric Institute, Kiwistraat 32, Den Haag, 2552 DH The Netherlands; 3 Department of TRANZO, Tilburg School of Social and Behavioral Sciences, Tilburg University, PO Box 90153, Tilburg, 5000 LE The Netherlands; 4 Faculty of Social and Behavioral Sciences, Institute of Psychology, Leiden University, Wassenaarseweg 52, Leiden, 2333 AK The Netherlands; 5 Yulius Mental Health, PO Box 1001, Dordrecht, 3300 BA The Netherlands; 6 ANTES Mental Health Care, Parnassia Psychiatric Institute, Albrandswaardsedijk 74, Poortugaal, 3172 AA The Netherlands

**Keywords:** Illness Management and Recovery, severe mental illness, psychosocial treatment, self-management, fidelity, recovery

## Abstract

Illness Management and Recovery (IMR) is a psychosocial intervention supporting people with serious mental illnesses. In this study, 15 IMR groups were assessed for fidelity and clinician competency to establish the implementation level of all IMR elements and explore complementarity of the IMR Treatment Integrity Scale (IT-IS) to the standard IMR Fidelity Scale. Use of the IT-IS was adapted, similar to the IMR Fidelity Scale. Descriptive statistics were applied. Implementation success of IMR elements varied widely on the IMR Fidelity Scale and IT-IS (*M* = 3.94, *SD* = 1.13, and *M* = 3.29, *SD* = 1.05, respectively). Twelve IMR elements (60%) were well-implemented, whereas eight (40%) were implemented insufficiently, including some critical cognitive-behavioral techniques (e.g., role-playing). The scales appeared largely complementary, though strongly correlated (*r* (13) = 0.74, *p =* 0.002). Providing all IMR elements adequately requires a variety of clinical skills. Specific additional training and supervision may be necessary.

## Introduction

Illness Management and Recovery (IMR) is a structured psychosocial intervention developed to support people with serious mental illnesses (SMI). IMR aims to provide support in coping with the physical, social, and emotional consequences of these illnesses (Lean et al., 2019; Mueser & Roe, 2016). In addition, it is designed to help people set and achieve personal goals. Thus, the overall objective of IMR is to facilitate recovery (Mueser et al., 2006). Based on an empirical review of the research literature concerning teaching illness self-management (Mueser et al., 2002a), five strategies were integrated into the IMR program: psychoeducation to promote knowledge of SMI and its treatment; behavior modification for medication adherence; relapse prevention training; social skills training to increase social support; and coping skills training for controlling persistent symptoms (Mueser et al., 2006).

IMR training consists of 11 modules, manuals, and handouts for the participants. The modules include recovery strategies, basic facts about mental illness, the stress vulnerability model, building social support, effective use of medication, drug and alcohol use, relapse reduction, coping with stress, managing persistent symptoms, meeting mental health needs, and maintaining a healthy lifestyle (Gingerich & Mueser, 2011). IMR trainers, i.e., clinicians providing IMR, apply three core teaching principles that should be used in every session (Meyer et al., 2010; Mueser et al., 2006). These include educational, motivational, and cognitive-behavioral strategies, which are applied in weekly IMR sessions lasting approximately one year. IMR can be conducted in a group or individually. In a group, the session structure includes all participants focusing on the topics of the modules; additionally, during each session, two or three participants follow up on their personal goals on a rotating basis (McGuire et al., 2016b; Meyer et al., 2010). IMR is currently used in several countries across North America, Europe, and Asia.

The IMR program is based on a combination of multiple evidence-based practices (EBPs) (Mueser et al., 2002a, 2003). However, as the results of seven randomized controlled trials (RCTs) on IMR are inconsistent, the effectiveness of the IMR program does not appear evident (Dalum et al., 2016, 2018; Fardig et al., 2011; Hasson-Ohayon et al., 2007; Jensen et al., 2019; Levitt et al., 2009; Roosenschoon et al., 2021; Salyers et al., 2014; Tan et al., 2017). In our recently published RCT, patients who were receiving IMR demonstrated statistically significant improved self-reported overall illness management (the primary outcome), as compared with usual care. In addition, they showed improvement in self-esteem, a component of personal recovery. No effects were found in other domains, including clinical and functional recovery. IMR completion was associated with stronger effects. In addition, high IMR fidelity was found to be associated with self-esteem (Roosenschoon et al., 2021). Altogether, in the mentioned RCTs on IMR, most evidence has been found in overall illness self-management, as measured by IMR scales (Fardig et al., 2011; Hasson-Ohayon et al., 2007; Levitt et al., 2009; Roosenschoon et al., 2021; Tan et al., 2017). Variations in results may be due to differences in patient population, sample size, type of control group, duration of IMR, levels of IMR exposure, and also differences in model fidelity (McGuire, Kukla, et al., 2014; Roosenschoon et al., 2016a, 2021).

Adequate dissemination and efficacy of research on EBPs for people with an SMI require clear and precise specification of the quality of implementation (Bond et al., 2009; Egeland, Heiervang, et al., 2019; Teague et al., 2012). Quality of implementation can be assessed by measuring the fidelity of interventions. Fidelity is the degree of adherence to standards and principles of a program model (Bond et al., 2009; Egeland, Heiervang, et al., 2019). Therefore, a primary reason why fidelity scales are developed is to evaluate whether an intervention is being implemented following the treatment model or program (model integrity). Relevant uses of assessing fidelity in research include determining whether the lack of effects of an intervention may be due to failure of proper implementation, comparing studies executed by different research groups and evaluating the same intervention, measuring differences in compliance to program standards over all sites in multi-site trials, and identifying critical ingredients of an intervention (Bond & Drake, 2020). The major relevance of fidelity lies in the assumption that “higher fidelity to an EBP predicts better outcomes for clients” (Bond & Drake, 2020). There is evidence of this predictive validity from some programs in the National EBPs Project in the US (McHugo et al., 2007), including Assertive Community Treatment (Latimer, 1999; McGrew et al., 1994; McHugo et al., 1999; Priebe et al., 2003; van Vugt et al., 2011), Individual Placement and Support (de Winter et al., 2020), and IMR (Hasson-Ohayon et al., 2007; McGuire et al., 2017; Roosenschoon et al., 2021).

The National EBPs Project aimed at evaluating the implementation of several different EBPs in typical mental health treatment programs in the U.S. Each EBP had a fidelity scale designed to evaluate the quality of implementation of that practice (McHugo et al., 2007). One of the programs in this project was IMR (Mueser et al., 2002a), for which the IMR Fidelity Scale was developed (Egeland, Heiervang, et al., 2019; McHugo et al., 2007). Later, another IMR fidelity scale was developed: the IMR Treatment Integrity Scale (IT-IS), which was meant to complement and extend the IMR Fidelity Scale by providing a more in-depth assessment of the trainer’s competence in applying IMR elements (McGuire et al., 2012). In this study, in 15 IMR groups of two mental health institutes for both scales, fidelity assessments were executed using the assessment procedure based on the IMR Fidelity Scale (McHugo et al., 2007).

In this study, we aimed to: (1) establish the level of implementation of all IMR elements, especially to identify targets for improvement; and (2) explore the complementary value of the IT-IS to the standard IMR Fidelity Scale. To the best of our knowledge, this is the first study in which the IMR Fidelity Scale and the IT-IS are compared at an item level.

## Methods

### Setting

This study was embedded in an RCT to test the effectiveness of IMR in people with SMI. This RCT was executed at two mental health organizations in the Rotterdam region of the Netherlands. Eligible participants were outpatients between 18 and 65 years of age who had been diagnosed with an SMI. IMR was provided in groups in weekly 90-minute sessions (Roosenschoon et al., 2016a, 2021).

Initially, based on knowledge obtained during a study tour to the US, an implementation plan was drafted for IMR implementation in both institutions. As part of this plan, a steering committee, implementation group, and education group were formed in each institution. The US handouts and IMR workbooks were translated into Dutch and edited for cultural relevancy. Moreover, in the larger of the two organizations, IMR implementation began with a pilot study that yielded positive results; an RCT seemed feasible (Roosenschoon et al., 2016b). After this pilot, IMR was integrated into the institutions’ standard care.

All IMR trainers participating in the RCT received two days of training in teaching IMR from two professional trainers who had extensive experience in teaching rehabilitation and recovery support as well as providing IMR. The IMR trainers received a two-hour group supervision from a senior counselor once every two weeks. Supervision groups were composed of a mix of IMR trainers from different IMR groups, teams, and locations. Twice a year, an additional four-hour training session was provided. Two master classes were held for trainers and counselors, each led by a US IMR creator.

A total of 35 IMR trainers were involved, who were experienced clinicians and had the following professional backgrounds: 15 community mental health nurses, four nurses, 13 social workers (four of whom were also peer support specialists), two psychologists, and one peer support specialist with a professional peer support education.

In this study, the IMR Fidelity Scale as well as the IT-IS were applied to 15 IMR groups. Ten groups had two IMR trainers, and five groups had three IMR trainers, one of whom was always a peer support specialist and also had a professional background as a clinician. On average, IMR groups with three trainers had more participants (*M* = 7.8, *SD* = 0.84) than those with two trainers (*M* = 6.2, *SD* = 1.48). However, this difference was statistically nonsignificant (U = 41, *p* = 0.06, *r* = 0.52). For this study, there were only limited changes in routine mental health care in each institution.

### Measures

The IMR Fidelity Scale is a scale used to assess the degree of implementation of the IMR model. It was part of the original IMR toolkit (Mueser et al., 2002b). It consists of 13 behaviorally anchored items that assess critical structural and clinical elements of IMR (McHugo et al., 2007). Structural elements include group size and program length, whereas clinical elements include the use of cognitive-behavioral techniques and coping skills training. Each item is rated on a five-point scale; a score of five indicates full implementation of the IMR element (Salyers et al., 2009). The total group score is the mean of all item scores. Scores of ≥ 4.0 are considered to reflect high fidelity, scores of ≥ 3.0 to < 4.0 reflect moderate fidelity, and scores of < 3 reflect low fidelity (McHugo et al., 2007). The IMR Fidelity Scale has shown excellent psychometric properties (Egeland, Heiervang, et al., 2019).

The IT-IS assesses trainer competence in conducting IMR elements displayed in a particular session and is also behaviorally anchored; treatment integrity is considered to include not only competence but also adherence to the IMR program and differentiation, that is, the flexible use of interventions (McGuire et al., 2012, 2016a, b, 2017). Competence—defined as IMR trainers’ level of skill shown in delivering the treatment—includes both understanding the program model and having the skills to implement it (McGuire et al., 2012). The IT-IS was meant to complement and extend the IMR Fidelity Scale by assessing individual clinicians. It provides researchers with a more precise assessment of the IMR process by evaluating competence at the clinical interaction level (McGuire et al., 2012).

The IT-IS consists of 16 items scored on a five-point scale, with higher scores indicating better performance. Guidelines for interpreting the scores are as follows: 1 to < 2 = *unsatisfactory*: clinician fails to use methods; ≥ 2 to < 3 = *needs improvement*: clinician applies either insufficient or inappropriate methods and/or with limited skill and flexibility; ≥ 3 to < 4 = *satisfactory*: clinician applies a sufficient range of methods with skill and flexibility, some difficulties evident; ≥ 4 to < 5 = *very good*: clinician systematically applies an appropriate range of methods in a creative, resourceful, and effective manner; 5 = *excellent*: clinician uses an excellent range of application or successful application in the face of difficulties. The total group score is the mean of all item scores (McGuire et al., 2016a, b).

Each of the IT-IS items corresponds to a critical element of IMR (McGuire et al., 2012). Four items are general, meaning that they are not specific to IMR but are critical to the quality of the intervention and include a therapeutic relationship, recovery orientation, involving all members of the group, and enlisting support among group members (McGuire et al., 2016b). The other 12 items are IMR-specific (McGuire et al., 2012). The IT-IS has 13 mandatory items and three optional items, which are only scored if the applicable part of the IMR curriculum is applied (i.e., coping skills training, relapse prevention planning, and behavior modification for medication); two items are only scored if IMR is taught in groups (i.e., involvement of group members and mutual support between group members) (McGuire et al., 2016b).

Unlike the IMR Fidelity Scale, the scoring system of the IT-IS includes so-called indicators of excellence, which reflect both characteristics of the use of the element (e.g., for recovery orientation, the provider maintains a “non-judgmental” attitude) or specific techniques (e.g., “shaping” for cognitive-behavioral techniques) (McGuire et al., 2016a). Often, the explanation of the indicators of excellence in IT-IS is quite detailed. For example, the explanation for shaping is “the reinforcement of *successive approximations* to a skill or a goal.” An example of one of the seven indicators of cognitive-behavioral techniques regarding cognitive restructuring is “helping the client describe the situation leading to the negative feeling, make a link between the negative emotions and the thoughts associated with those feelings, evaluate the accuracy of those thoughts, and, if they are found to be inaccurate, identify an alternative way of looking at the situation that is more accurate” (McGuire et al., 2016b). Another example is that for motivational enhancement strategies, indicators of excellence are listed concerning seven IMR principles: evocation, development of discrepancy, collaboration, autonomy/support, rolling with resistance, direction, and empathy. Moreover, for each principle, examples of proficient execution and a violation are provided (McGuire et al., 2016b).

More than the IMR Fidelity Scale, the IT-IS protocol indicates that specific aspects of an element must be present in practice for it to achieve certain scores. For example, “note that the client must have completed the action plan for there to be evidence for the last two indicators: the action plan integrated material into their recovery and goals, and had an obvious positive effect on recovery” (in action plan follow-up) (McGuire et al., 2016b). In scoring, raters should even penalize for missed opportunities to apply cognitive-behavioral techniques (McGuire et al., 2016b). Therefore, it is the presence or absence of a clinician’s behavior that must be scored.

Psychometric analysis of the IT-IS has demonstrated excellent inter-rater reliability, good factor structure validity, and acceptable internal consistency and discriminant validity (McGuire et al., 2012).

### Procedures

The principal investigator and one of the two co-auditors conducted IMR fidelity assessments during one-day visits to each of the 15 IMR groups. Both assessors scored independently following a standard procedure and manuals (McHugo et al., 2007). In case of differences, the evaluators determined a consensus score. Because groups had more than one IMR trainer, ratings were based on how the trainers functioned together (McGuire et al., 2016b). The principal investigator was trained by two US experts at assessing IMR fidelity (Roosenschoon et al., 2016a). The two educated co-auditors were a psychologist and an advanced nurse practitioner, both of whom had experience in providing IMR themselves. Besides semi-structured interviews with IMR participants and trainers of the particular IMR group, assessments consisted of one observational session and monitoring of forms, such as anonymous IMR Goal Tracking Sheets and progress notes regarding the five latest IMR sessions. To get a complete picture, IMR fidelity assessments were conducted during one of the last sessions of the curriculum. This was on average about a year after the IMR trainers’ initial training. During the interviews, all IMR elements were reviewed with all respondents.

While the applied assessment procedure was based on the IMR Fidelity Scale protocol (McHugo et al., 2007), the IT-IS was designed to rate the fidelity of clinicians to the IMR program based on observations of treatment sessions (either live, audio recorded, or video recorded) (McGuire et al., 2016a, b). However, the logistics of organizing multiple recordings per IMR group and subsequently assessing them were not feasible in the context of this RCT. Therefore, in this study, the IT-IS rating was adapted based on the IMR Fidelity Scale. Interviews for the IMR Fidelity Scale were extended to allow for the rating of the additional items in the IT-IS. Moreover, using the indicators of excellence, the rating of the IT-IS items required detailed questioning on the application of various competencies; this included questions on competencies that were not or hardly used during the observed session. These interviews aimed to capture IMR practitioners’ knowledge and actual skill level in the concrete application of the various aspects of the IMR competencies specified in the IT-IS protocol for each item. This also included requesting examples. For both scales per IMR group, various participants’ responses to the same question were continuously checked for consistency. Furthermore, the observations and chart review results were always the starting point for the interviews. For all elements, information from direct observations, interviews, and chart reviews was integrated by the assessors to score the fidelity scales. Thus, a good overall picture of the fidelity within each group was obtained, and assessing all items of the IMR Fidelity Scale and the IT-IS could be achieved. With this fidelity data collection method, we could minimize the potential impact of which particular IMR session was observed. Moreover, previous research on the IT-IS showed the limited impact of the module covered in the rated session (McGuire et al., 2016a).

The clinicians leading the groups and the team leaders received a report on the scores in their group. Subsequently, researchers provided fidelity feedback on the aggregated study results two or three times per supervision group up to that point. Subsequently, recommendations for the improvement of poorly implemented elements were discussed, as were ways to achieve these improvements. In addition, this fidelity feedback was provided in plenary sessions for all stakeholders per institution.

The IMR groups were ranked by total scores on both the IMR Fidelity Scale and the IT-IS. A high group score on the IMR Fidelity Scale was defined as ≥ 4. Because the IT-IS has no predefined cut-off for group-level total scores, we used the cut-off scores at the item level for this purpose: 1 to < 2: *unsatisfactory*; ≥ 2 to < 3: *needs improvement*; ≥ 3 to < 4: *satisfactory*; ≥ 4 to < 5: *very good*; 5: *excellent*.

For both the IMR Fidelity Scale and the IT-IS, items were ranked across all groups in descending order of mean scores to explore which critical elements were implemented well and which were not. In addition, for the IMR Fidelity Scale per item, the percentage of groups with high scores was calculated. A high item score was defined as ≥ 4, a moderate item score as ≥ 3.0 to < 4.0, and a low item score as < 3. For the IT-IS per item, the percentage of groups with scores of *satisfactory* and higher was calculated.

### Analysis

First, we described which IMR elements were measured by each of the two instruments. Second, descriptive statistics per IMR group were utilized to investigate the overall IMR fidelity (mean score of the IMR Fidelity Scale) and competency (mean score of the IT-IS). Third, descriptive statistics per IMR element and scale were utilized to investigate item-level fidelity. Fourth, the IT-IS indicators of excellence were used to examine the elements scored in the *needs improvement* and the *unsatisfactory* range in detail.

Pearson’s correlation coefficient was used to measure the association between total scores of the IMR Fidelity Scale and the IT-IS. Three categories were applied in our interpretation of correlations: weak (0.1, 0.3), moderate (0.3, 0.5), and strong (0.5) (Cohen, 1988). SPSS 27 was used for data analysis.

The study protocol was approved by the accredited medical ethics trial committee at Erasmus University Medical Center Rotterdam (27/8/2012, METC nr NL38605.078.12) and is registered in the Netherlands Trial Register NL4931 (NTR5033). The authors declare that they have no conflicts of interest.

## Results

### IMR Elements Assessed with Each Scale

There appeared to be considerable overlap because eight IMR elements were measured using both scales. However, five elements were only assessed by the IMR Fidelity Scale, and eight elements were only assessed by the IT-IS (Table 1).

**Table 1:** IMR-elements assessed with the IMR fidelity scale and the IMR Treatment Integrity Scale (IT-IS)

Elements assessed with both scales	Elements assessed only with the	Elements assessed only with the
	IMR fidelity scale	IT-IS
Comprehensiveness of the Curriculum	Program Length	Weekly Action Planning
Educational Techniques	Provision of Educational Handouts	Action Plan Review
Motivation-Based Strategies	Number of People in a Session or Group	Structure/Efficient Use of Time
Relapse Prevention Training	IMR Goal Setting	Involving All Members of the Group
Coping Skills Training	IMR Goal Follow-up	Recovery Orientation
Cognitive-Behavioral Techniques		Therapeutic Relationship
Behavioral Tailoring for Medication		Enlisting Support Between Group Members
Involvement of Significant Others		Goals

The eight elements assessed by both scales included seven clinical elements and one structural element. The five elements assessed only by the IMR Fidelity Scale included three structural elements and two clinical elements related to IMR participants’ personal goals. The eight clinical elements assessed only by the IT-IS involved four so-called general items and four IMR-specific elements. The IT-IS item “goals” combined the items “IMR goal-setting” and “IMR goal follow-up” from the IMR Fidelity Scale. Therefore, in total, 20 different IMR elements were included.

### Results for the IMR Fidelity Scale

#### High- and Low-Scoring IMR Groups

Fidelity assessment for the IMR Fidelity Scale of participants in 15 IMR groups produced an average moderate group score of 3.94 (*SD* = 0.29). Eight groups (53%) had total scores of ≥ 4 (range 4.00–4.54), indicating high fidelity, and seven groups (47%) had total scores of ≥ 3 and < 4 (range 3.46–3.92), indicating moderate fidelity (McHugo et al., 2007).

#### High- and Low-Scoring Elements

The degree of implementation success of the numerous fidelity IMR elements varied widely (*M* = 3.94, *SD* = 1.13) (Table 2). In Table 2, the IMR Fidelity Scale items are ranked across all groups in descending order of mean scores per item. This table shows a division above and below the cut-off score of 4 for seven high-scoring items (range of mean scores: 4.60–5.00), five moderate-scoring items (range of mean scores: 2.50–3.53), and one low-scoring item (1.80).

The six moderate- and low-scoring items all involved clinical elements of IMR. Out of these, three elements were poorly implemented by nine groups (60%), including cognitive-behavioral techniques, IMR goal follow-up, and relapse prevention training. Three other elements were poorly implemented by 12–14 IMR groups (80–93%), including the involvement of significant others, coping skills training, and behavioral tailoring for medication.

**Table 2:** Implementation of critical elements in 15 IMR-Groups rated on the IMR-fidelity scale

Item	*M*	*SD*	Min	Max	% of groups with high item rating^a^
Items with average rating of ‘high’					
Program Length	5,00	0,00	5,00	5,00	15 (100%)
Provision of Educational Handouts	5,00	0,00	5,00	5,00	15 (100%)
Comprehensiveness of the Curriculum	4,93	0,26	4,00	5,00	15 (100%)
Educational Techniques	4,93	0,26	4,00	5,00	15 (100%)
Number of People in a Session or Group	4,87	0,35	4,00	5,00	15 (100%)
Motivation-Based Strategies	4,67	0,62	3,00	5,00	14 (93,3%)
IMR Goal Setting	4,60	0,63	3,00	5,00	14 (93,3%)
Items with average rating of ‘moderate’					
Cognitive-Behavioral Techniques	3,53	0,74	3,00	5,00	6 (40,0%)
IMR Goal Follow-up	3,47	1,13	2,00	5,00	6 (40,0%)
Relapse Prevention Training	3,38	0,97	2,00	5,00	6 (40,0%)
Behavioral Tailoring for Medication	2,60	0,71	1,00	4,00	1 (6,7%)
Coping Skills Training	2,50	0,73	2,00	4,00	2 (13,3%)
Item with average rating of ‘low’					
Involvement of Significant Others	1,80	1,37	1,00	5,00	3 (20,1%)
Total	3,94	1,13			

^a^ The number (%) of IMR groups with a high rating of ≥ 4 on this item.

### Results for the IT-IS

#### High- and Low-Scoring IMR Groups

Fidelity assessment for the IT-IS of participants in 15 IMR groups yielded an average total competence score of satisfactory (*M* = 3.29, *SD* = 0.29; range: 2.88–3.88). All groups except one (93%) had a satisfactory total score (range: 3.00–3.88). One group (7%) had an *unsatisfactory* total score of 2.88.

#### High- and Low-Scoring IMR Elements

The degree of implementation success of the various competence elements of IMR varied widely (*M* = 3.29, *SD* = 1.05). In Table 3, the items of the IT-IS are ranked across all groups in descending order of mean scores per item. This table shows a division above and below the cut-off score of 3 for nine *satisfactory* and higher scoring items (range of mean scores: 3.13–4.87), six *needs improvement* scoring items (range of mean scores: 2.07–2.83), and one *unsatisfactory* scoring item (*M* = 1.73).

**Table 3:** Implementation of critical elements in 15 IMR-groups rated on the IMR Treatment Integrity Scale (IT-IS)

Item	*M*	*SD*	Min	Max	% of IMR-groups with ≥ satisfactory item rating^a^
Items with average rating of very good					
Use of Structured IMR Curriculum	4,87	0,35	4,00	5,00	15 (100%)
Educational Strategies	4,67	0,49	4,00	5,00	15 (100%)
Therapeutic Relationship	4,60	0,51	4,00	5,00	15 (100%)
Involving All Members of the Group	4,47	0,64	3,00	5,00	15 (100%)
Recovery Orientation	4,13	0,52	3,00	5,00	15 (100%)
Items with average rating of satisfactory					
Enlisting Support Between Group Members	3,93	0,59	3,00	5,00	15 (100%)
Goals	3,47	1,25	2,00	5,00	11 (73,3%)
Motivational Enhancement Strategies	3,40	0,83	2,00	5,00	13 (86,7%)
Structure/Efficient Use of Time	3,13	0,92	2,00	5,00	12 (80,0%)
Items with average rating of needs improvement					
Relapse Prevention Training	2,83	1,06	2,00	5,00	6 (40,0%)
Behavioral Tailoring for Medication	2,52	0,72	1,00	4,00	7 (46,7%)
Weekly Action Planning	2,40	0,83	1,00	4,00	7 (46,7%)
Coping Skills Training	2,28	0,59	1,00	3,00	5 (33,3%)
Cognitive-Behavioral Techniques	2,13	0,35	2,00	3,00	2 (13,3%)
Action Plan Review	2,07	0,88	1,00	3,00	6 (40,0%)
Item with average rating of unsatisfactory					
Involvement of Significant Others	1,73	1,10	1,00	5,00	2 (13,3%)
Total	3,29	1,05			

^a^ The number (%) of IMR groups with at least a satisfactory rating of ≥ 3 on this item.

The seven *needs improvement* and *unsatisfactory* scoring IT-IS items all involved clinical elements of IMR. Out of these seven elements, five were poorly implemented by between eight and ten IMR groups (53.3–66.7%), including behavioral tailoring for medication, weekly action planning, relapse prevention training, action plan review, and coping skills training. Two elements—the involvement of significant others and cognitive-behavioral techniques—were poorly implemented by 13 groups (86.7%).

#### Results on the Indicators of Excellence of the Poorly Implemented Elements

To better comprehend and clarify the poor application of the seven *needs improvement* and *unsatisfactory* scoring competency elements, we investigated the results of the application of the indicators of excellence, which provided direction to the rating of these elements (McGuire et al., 2016a).

IMR trainers did not make *individual relapse prevention plans* with all the participants, nor did they check existing plans. In addition, they did not succeed in persuading participants to try out components of the plan or ensuring that all people involved were familiar with it. For *behavioral tailoring for medication*, after thorough discussion, IMR trainers should help participants find individual ways to incorporate taking medication into their daily lives and discuss medication use with their physician. However, this element was often misunderstood as only promoting the exchange of experiences with different types of medication and their side effects. For *coping skills training*, none of the IMR groups systematically used an appropriate set of methods, including role-play, modeling, or shaping. In addition, encouraging significant others to participate in coping strategies was rare. *Cognitive-behavioral technique* reinforcement was often used. However, only some groups used relaxation training and occasionally modeling. Role-play, shaping, cognitive restructuring, and behavioral experiments were not applied. Most IMR trainers only gave general assignments to the group instead of *weekly individual action planning and review*. Weekly action planning refers to assignments to help client(s) transfer skills presented during the session to their daily lives and also includes steps to be taken to attain measurable benchmarks of goal progress (McGuire et al., 2016b). However, the trainers rarely helped individual participants’ tailor their planned activities for their goals, preferences, and personal situations. Consequently, there were few reviews of personal action plans. Finally, the lowest average score was on *significant other involvement competence*. In almost no group did IMR trainers have a plan to systematically increase the involvement of significant others, for example, in homework or in working on recovery goals or by having them attend an IMR session.

### Two Examples of Successful Implementation of the Most Poorly Scored Element

Although a total of eight elements scored poorly on average, some IMR groups managed to implement these elements with *high* or *satisfactory* scores. Generally, scores ranged from 2 to 3 points on the five-point-scales (Tables 2 and 3). Two examples of successful implementation of the element of significant other involvement that had the lowest score on both scales are as follows. One IMR trainer elicited involvement by explaining IMR to the families of two of her clients, who were also in the IMR group. Consequently, one participant had a goal of reading to her children. In the highest-scoring group for this element, trainers frequently asked to involve significant others in IMR activities based on a specific plan. In this group, some participants and their partners completed home assignments together. When the social support module was discussed in this group, each participant was invited to bring someone with some success, such as a housing support worker, sister, wife, and so on. Therefore, in this IMR group, the trainers managed to meet the IT-IS excellence indicator of having a family member physically present at a session.

## Discussion

### Study Relevance and Main Results

To the best of our knowledge, this study is the first to use both the IMR Fidelity Scale and the IT-IS to identify the degree of IMR implementation per group and at the item level. In addition, this study evaluated the complementary value of the IT-IS to the IMR Fidelity Scale, including the shared and separate elements covered by the two scales. For use in this study, the IT-IS rating procedure was adapted so it could be used in a way similar to the IMR Fidelity Scale.

Results showed that 12 of the 20 IMR elements of both scales (60%) were well-implemented (use of a structured IMR curriculum, educational strategies, motivation-based strategies [all three assessed with both scales], provision of educational handouts, comprehensiveness of the curriculum, number of people in a session or group, IMR goal-setting [all four assessed with the IMR Fidelity Scale], therapeutic relationship involving all members of the group, recovery orientation, enlisting support between group members, and structure/efficient use of time [all five assessed with the IT-IS]). Eight IMR elements were insufficiently implemented (involvement of significant others, cognitive-behavioral techniques, behavioral tailoring for medication, relapse prevention training, coping skills training [all five assessed with both scales], IMR goal follow-up [only assessed with the IMR Fidelity Scale], weekly action planning, and action plan review [only assessed with the IT-IS]). These results suggest that both scales should be used to obtain a complete picture of IMR implementation.

In implementing IMR, three core teaching principles (motivational, educational, and cognitive-behavioral strategies) should be used every session by the trainers to enable skills training for promoting illness self-management (Meyer et al., 2010; Mueser et al., 2006). Although motivational and educational strategies were sufficiently implemented in this study, cognitive-behavioral strategies were not. The implementation of all eight poorly implemented IMR elements especially requires competence in the cognitive-behavioral techniques of role-playing, modeling, and using home assignments (Meyer et al., 2010). However, these techniques were rarely applied.

### Relative Value of the IT-IS to the Standard IMR Fidelity Scale

Both instruments aim to assess IMR fidelity. Regarding the different IMR elements assessed by both scales, there is considerable overlap because eight IMR elements are measured by both scales. However, five elements are only assessed by the IMR Fidelity Scale, and eight other elements are only assessed by the IT-IS. There is more overlap because the IT-IS item “goals” combines the items “IMR goal-setting” and “IMR goal follow-up” from the IMR Fidelity Scale. However, separate scoring of these items in the IMR Fidelity Scale seems beneficial, as it is practically relevant to know if “goal-setting” is better realized than “goal follow-up,” as it was in the current study.

However, despite the observed overlap and high correlation, the description and operationalization of the items, as well as the focus of both scales, are quite different. The IMR Fidelity Scale is considered a more global program-level measure (McGuire, Luther, et al., 2014), while the IT-IS is a more detailed clinician-level measure. The more detailed operationalization of the IT-IS items appears to have added value because it provides precise direction for scoring and therefore may be used for advanced training and supervision. Furthermore, the IMR Fidelity Scale was developed much earlier (Mueser et al., 2002b) and is used more often, which improves comparability across studies (Bond & Drake, 2020). To date, in four of the RCTs on IMR, results for the IMR Fidelity Scale were reported (Dalum et al., 2018; Hasson-Ohayon et al., 2007; Jensen et al., 2019; Levitt et al., 2009; Roosenschoon et al., 2021); and in two RCTs on IMR, the IT-IS was applied (Roosenschoon et al., 2021; Salyers et al., 2014).

In this study, we found that the mean overall score of the IT-IS was 0.65 (16%) lower than the mean overall score of the IMR Fidelity Scale. This appears to be partly due to the IT-IS’s greater focus on clinical competency elements, which are harder to achieve than structural elements. In addition, the IT-IS incorporates more operationalizations and conditions to fulfill, including indicators of excellence. Therefore, we suggest that the IT-IS scale is largely complementary to the IMR Fidelity Scale but also more rigorous in its assessment of implementation quality.

In this study, we found that the extended operationalizations of the IT-IS provided precise direction for needed additional IMR implementation support. However, in our view, the addition of the three missing structural items of the IMR Fidelity Scale (program length, provision of educational handouts, and number of people in a session or group) would make the IT-IS more complete. Furthermore, splitting the IT-IS “goals” item into “goal-setting” and “goal follow-up,” as in the IMR Fidelity Scale, might also improve the usability of the IT-IS.

The rating procedure of the IT-IS in this study was different from the original intent because extended interviews with IMR participants and trainers of each IMR group and chart reviews were employed to review, per IMR element, the specific behavioral characteristics for clinician competence in sessions in addition to the ones observed. By using this method for collecting fidelity data, we suggest that we were able to attain the required specificity.

Both scales address fidelity through adherence to the IMR model (Bond et al., 2009; Heiervang et al., 2020; McGuire et al., 2012). To this end, both scales address more clinical than structural elements. We suggest that clinical elements address clinical competency. Therefore, it appears that both scales aim to address fidelity more through the clinical competence of IMR trainers than through the application of structural IMR elements.

### Comparing Results with Other Studies

In this study, assessed with the IMR Fidelity Scale, average fidelity was slightly lower than the weighted mean of six studies reported in an IMR review (*M* = 4.05, *SD* = 0.93) (McGuire, Kukla, et al., 2014). This might be partly due to the relatively high degree of refinement of the applied assessment procedure because, in this study, the assessment procedure included all components of the protocol (McHugo et al., 2007; Salyers et al., 2009). Measured using the IT-IS, the average fidelity was also lower than that in one RCT (Salyers et al., 2014). However, it was markedly higher than in another study on implementing IMR in community practice (McGuire et al., 2016a).

Our results regarding the implementation levels of the different elements measured with the IT-IS are largely in line with the results of one previous study (McGuire et al., 2016a). Although fidelity in this earlier study was lower overall, the ranking was similar: the five elements with the lowest fidelity—medication management, weekly action planning, action plan follow-up, cognitive-behavioral techniques, and significant other involvement—were among the seven elements with the lowest fidelity in the present study (McGuire et al., 2016a).

In another study, results regarding the lower scoring items on the IT-IS were also quite similar (McGuire et al., 2012). However, in the overview of elements in this study, data for the three optional items were excluded. The similarities between the results of these three studies appear to support generalization. This provides direction for improvement efforts in the future. However, it should be noted that comparing the IT-IS ratings in this study with those from previous research that also used this scale should be done with caution considering differences in how the instrument was used.

### Relevance and Interrelationship of the Poorly Implemented Elements

Providing IMR requires mastery of a variety of advanced clinical skills (McGuire et al., 2016a). In our view, the reasons for the poor implementation of the eight IMR elements might be interrelated, as most of these elements involve cognitive-behavioral skills. This is outlined in the following two sections, using a guide to IMR implementation (Meyer et al., 2010).

Setting and follow-up on personal goals are central elements of IMR (McGuire et al., 2012, 2016a; Meyer et al., 2010; Mueser et al., 2006). At the start of the curriculum in IMR Module 1, individual goals are set to work on the IMR training. Supporting participants in *following up on goals* (1) by using goal charts should be a routine part of every IMR session (Meyer et al., 2010). This goal follow-up involves *action planning* (2), wherein goals are broken down into smaller, intermediate goals, allowing one to work step by step toward achieving the goals as *home assignment*s between sessions. This facilitates the evaluation of progress in the *action plan review* (3) every session. Achieving personal goals is more successful when there is close cooperation among participants, trainers, and *significant others* (4) (Meyer et al., 2010). In *coping skills training* (5), after *modeling* by the trainer, newly selected coping strategies are practiced during the IMR session. Subsequently, a *home assignment* is developed with the participant to practice the coping skill independently (Meyer et al., 2010; Tarrier, 1992). This may also include coping skills training to respond to a trigger of relapse or early warning signs, as part of the implementation of a *relapse prevention plan* (6). In such a plan, *significant others* may be quite important to include because they may help identify those signs and triggers. *Home assignments* regarding taking medications may be linked to participants’ *recovery goal*. It may include reviewing the benefits and side effects of medications with a *significant other*. If a participant has decided to take medication, they could make an *action plan* to use *behavioral tailoring of medication* (7) or practice talking to the doctor in a *role-play*. Central to the implementation of all these IMR elements should be the use of *cognitive-behavioral techniques* (8), especially techniques for behavioral rehearsal, mainly role-playing (Meyer et al., 2010). Application of *cognitive-behavioral techniques* includes one of the three core teaching principles that should be used by the IMR trainers every session to enable skills training for promoting illness self-management (Meyer et al., 2010; Mueser et al., 2006). In the current study, motivational and educational strategies were sufficiently implemented; however, cognitive-behavioral strategies were not.

IMR participants, by practicing a skill both inside and outside of a session, will feel more confident in using that skill in everyday life. Role-playing provides the trainer with a structure for practicing a skill using interactive teaching methods. Teaching a skill using role-play can often be combined with modeling, which can be executed by the IMR trainer (Meyer et al., 2010). Home assignments are also a cognitive-behavioral technique (Meyer et al., 2010). In the implementation guide, these cognitive-behavioral techniques were consistently named as critical ingredients for the successful implementation of the poorly implemented elements in this study (Meyer et al., 2010). Therefore, we suggest that the poor implementation of these critical cognitive-behavioral techniques may have greatly impeded the implementation of the other seven insufficiently implemented elements.

Role-playing was completely unused in the IMR groups of this study. However, role-play appears to be a crucial component of IMR implementation (Meyer et al., 2010). IMR trainers should be familiar with it because it has been used in their education. However, in the interviews with both trainers and clients, it appeared that before IMR, both groups had experienced anxiety and stage fright with role-playing. Therefore, in implementing IMR, the trainers felt uncomfortable about doing role-play, partly due to anticipated reluctance from clients. This resulted in avoidance.

In addition, they appeared to avoid giving homework assignments for fear that this would lead to dropout. The lack of readiness to use role-plays, modeling, and home assignments would appear to be explained as a shortcoming in the training process of IMR trainers. The IMR trainers’ discomfort could be overcome by practicing these skills regularly during training and supervision sessions. Therefore, improvements in the training of IMR clinicians could address these common concerns of practitioners and better equip them with the cognitive-behavioral skills they require to effectively implement the IMR program.

Some relevant suggestions from the implementation guide mentioned above were not applied (Meyer et al., 2010), for example, using experiential learning exercises in the initial two-day training for IMR trainers and specialized follow-up training in motivational and cognitive-behavioral strategies. In addition, IMR implementation was not initiated with a very small group of participants to practice, and supervision was provided only once every two weeks instead of once a week. One might suggest that more psychologists should be employed as IMR trainers. However, a previous study indicated that mental health professionals from diverse backgrounds were able to apply IMR with high fidelity, though sufficient training and continuous supervision were considered critical (Garber-Epstein et al., 2013).

Therefore, more specialized training and experience in these skills were required so that using role-play, modeling, and home assignments could become a routine activity at the sites participating in the current study.

### Implications of this Study

In addition to these relevant cognitive-behavioral techniques, the current study showed serious shortcomings in IMR trainers’ adherence to the IMR model. These shortcomings were also shown in other defining IMR characteristics in more than half of the IMR groups each time. The IMR trainers concerned did not routinely follow up on goals set by participants, did not teach participants relapse prevention or coping skills, and did not incorporate behavioral tailoring to improve medication self-management.

One practical approach would be applying the technique of systematically using fidelity-based feedback to shape IMR clinicians’ clinical skills (Bond et al., 2009; Lu et al., 2012). This means that training and supervision of clinicians in IMR should include information on clinicians’ fidelity to the model, collected on a routine basis, preferably using objective methods (such as completing the IT-IS based on audio tapes of sessions). Using this information, directions can be provided for training specific competencies of clinicians to improve fidelity to the model.

Originally, the primary evidence base for the development of the IMR program included research demonstrating the beneficial effects of cognitive-behavioral techniques, behavioral tailoring for medication, relapse prevention training, and coping skills training (Mueser et al., 2002a). In the Introduction, it was mentioned that one of the causes for variations in the results of the RCTs on IMR might have been differences in model fidelity (McGuire, Kukla, et al., 2014; Roosenschoon et al., 2016a, 2021). Therefore, poor fidelity to these IMR elements could have contributed to some of this variability in outcomes, including the lack of effects on symptomatic or functional outcomes other than illness self-management found for IMR in our own RCT (Roosenschoon et al., 2021).

### Strengths and Limitations

To our knowledge, this is the first study to examine both IMR fidelity and IMR clinician competence at the group and item levels using the IMR Fidelity Scale and the IT-IS. This helped determine the level of implementation of IMR elements and identify poorly implemented elements. This knowledge can be used for directing the training and supervision of IMR trainers to improve the quality of IMR implementation. In addition, we were able to determine the relative value of the IT-IS to the standard IMR Fidelity Scale.

During the execution of this study, IMR had become a part of routine mental healthcare in both institutions. Therefore, the results may be generalizable to other sites. Generalization might also be supported by the similarities in outcomes between this study and two other studies that examined IMR fidelity at the item level (McGuire et al., 2012, 2016a). However, comparing the IT-IS ratings in this study with those from previous research that also used this scale should be done with caution considering differences in how the instrument was used.

In addition to other elements, both scales measure eight of the same IMR elements. The two scales were scored sequentially per assessor. Therefore, scoring on two scales of the same IMR elements could have influenced each other. However, the elements were scored with the two scales from a different perspective. Moreover, two different manuals were applied, and scores were discussed separately for each scale by the two assessors.

The rating of both scales may have been limited by only including one observational session. However, the manual of the IMR Fidelity Scale specifies that after interviews and chart review, only one session must be observed. Conversely, the IT-IS rating should be based on observations of IMR sessions (live or based on audio or audiovisual recordings). Therefore, in this study, we adapted the IT-IS rating procedure based on the IMR Fidelity Scale rating procedure and used extended interviews. We thus suggest the results of this study are relevant.

Finally, realization of organizational conditions is critical to successful IMR implementation (Egeland, Hauge, et al., 2019; Heiervang et al., 2020; Teague et al., 2012; van Weeghel, 2020). In this study, however, we chose to focus on adherence to the IMR model and the fidelity of the competencies of IMR trainers.

## Conclusions

Adequate fidelity in IMR implementation is important for many reasons, one of which is the impact on IMR outcomes. Proper IMR implementation requires trainers with a broad set of advanced knowledge and specific clinical skills. The majority of IMR elements appeared sufficiently implemented. However, for most IMR trainers, eight relevant IMR elements regarding clinical skills were found to be difficult to implement. Some cognitive-behavioral skills, especially using role-play, modeling, and home assignments, are critical for implementing these elements. Therefore, IMR trainers who lack skills across these eight elements may need supplementary training following the initial training in IMR to teach cognitive-behavioral skills (e.g., a one- or two-day workshop). Skills would also be effectively reinforced by the routine incorporation of training in supervision sessions. Furthermore, the need for training in specific cognitive-behavioral skills could be determined by systematically providing feedback on clinician fidelity ratings obtained during the course of their providing the program, such as with the IT-IS based on audio recordings of IMR sessions.
